# Supplementary material for: DMRforPairs: identifying Differentially Methylated Regions between unique samples using array based methylation profiles
Source: BMC Bioinformatics. 2014 May 15;15:141. doi: 10.1186/1471-2105-15-141 (PMC4046028; doi:10.1186/1471-2105-15-141)
Supplement: Additional file 2 — DMRforPairs output for the comparison of A431-MCF7 and NA17018-NA17105. Please start from the HTML files in each folder. Available via the BMC Bioinformatics website. [file 1471-2105-15-141-S2.zip › 1394847754114233_MOESM2_ESM/A431_MCF7/figures/33894.pdf]

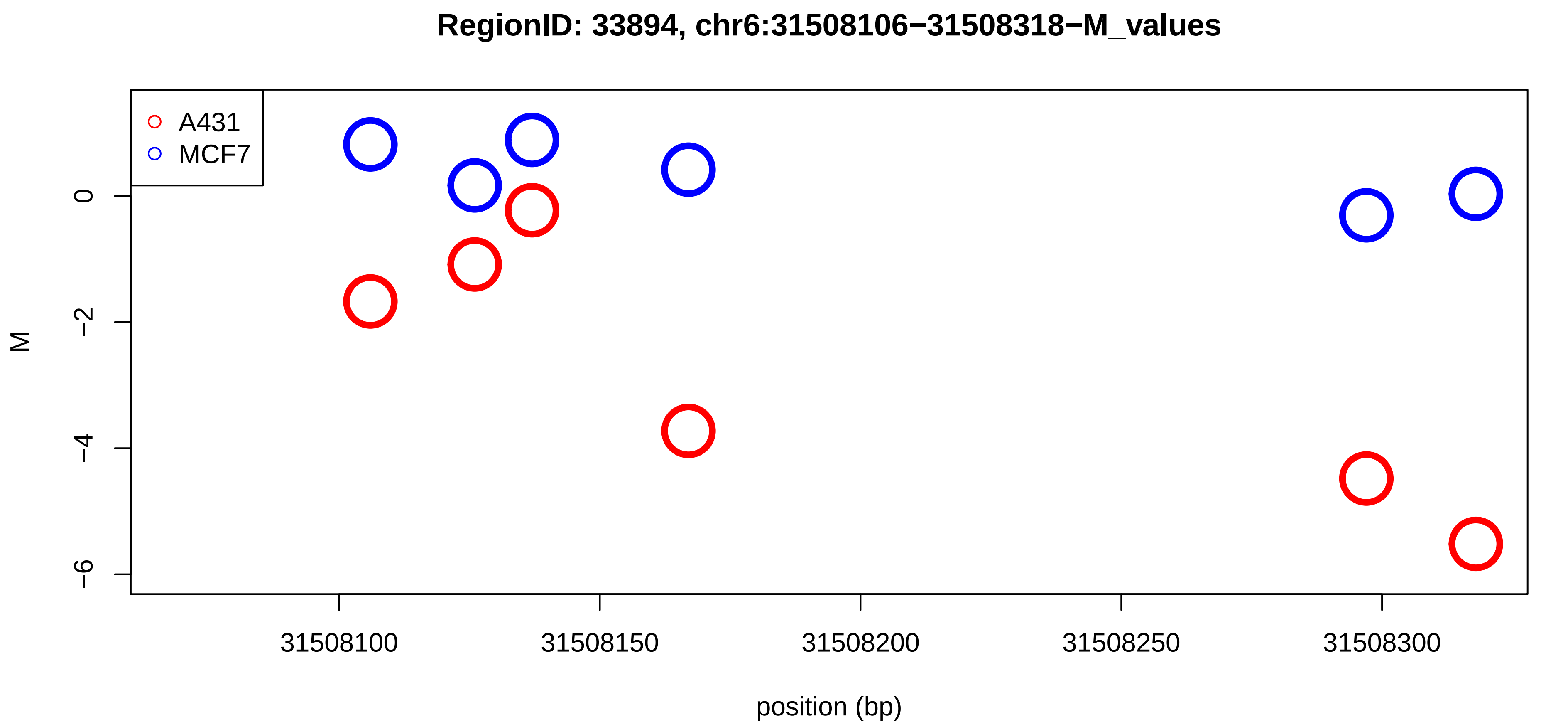

RegionID: 33894, chr6:31508106−31508318−M_values
A431
MCF7
M
0
−2
−4
−6
31508100
31508150
31508200
31508250
31508300
position (bp)

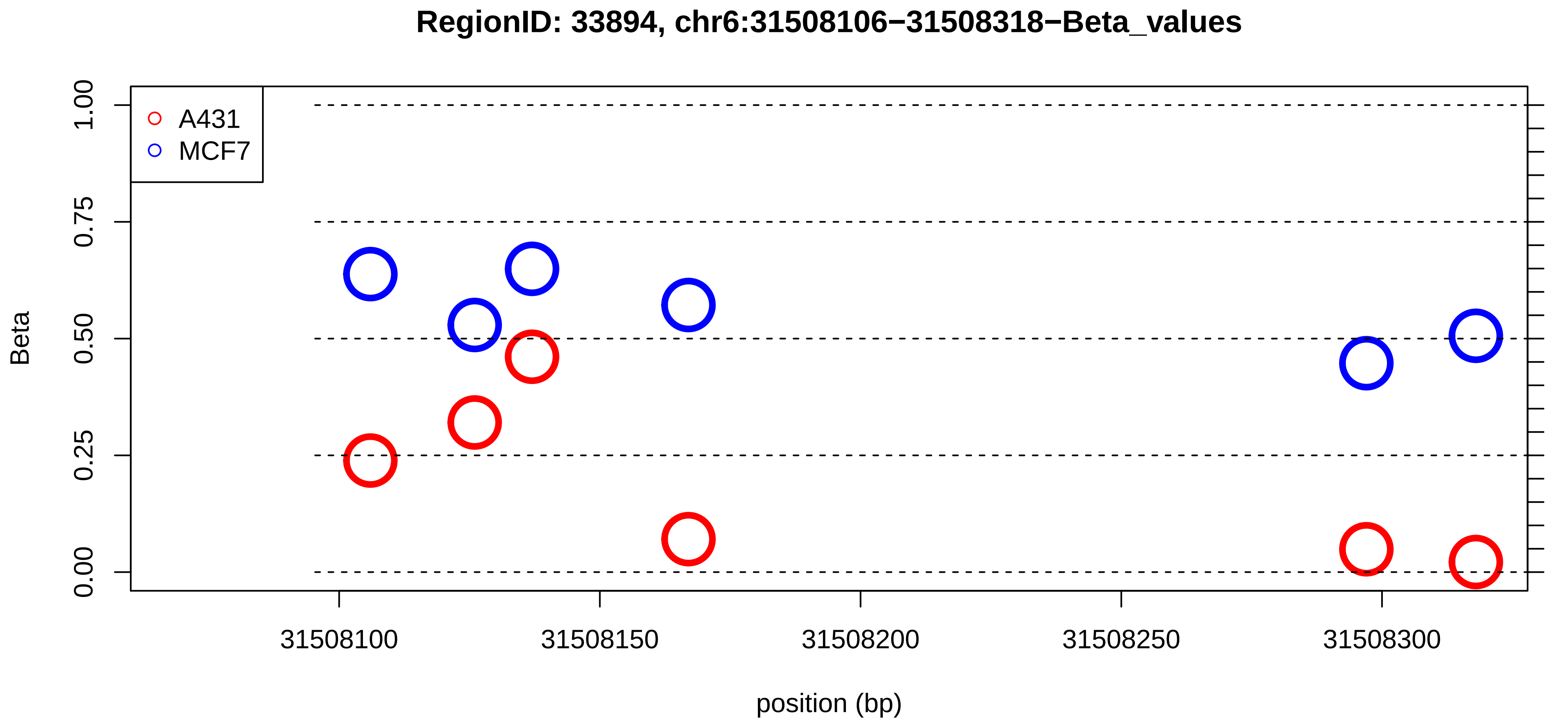

RegionID: 33894, chr6:31508106−31508318−Beta_values
A431
MCF7
Beta
1.00
0.75
0.50
0.25
0.00
31508100
31508150
31508200
31508250
31508300
position (bp)

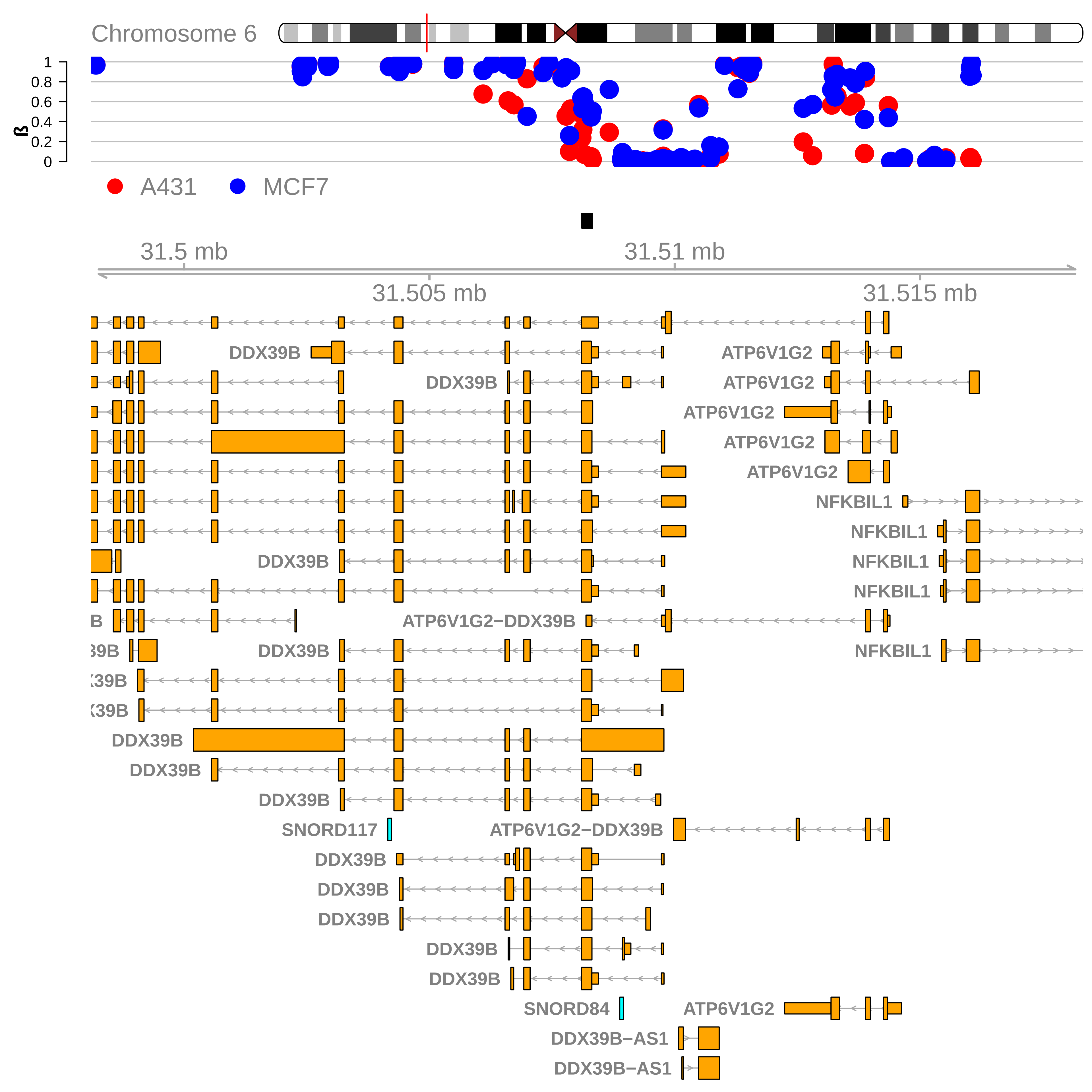
Chromosome 6
ß
1
0.8
0.6
0.4
0.2
0
A431
MCF7
31.5 mb
31.51 mb
31.505 mb
31.515 mb
DDX39B
ATP6V1G2
NFKBIL1
ATP6V1G2−DDX39B
SNORD117
SNORD84
DDX39B−AS1
